# Cytotoxic Activities of O-Cholesteryl-O-Phenyl-N-Phenylphosphoramidate and Its Organometallic Tin(lV) Derivatives

**DOI:** 10.1155/MBD.2002.333

**Published:** 2002

**Authors:** Marcela López-Cardoso, Patricia García y García, Raymundo Cea-Olivares, María- Luisa Villareal

**Affiliations:** 1 Center of Chemical Research. Autonomous University of Morelos Av. Universidad 1001 Cuernavaca Morelos 62210 Mexico; 2 Institute of Chemistry National Autonomous University of México Circuito Exterior, Ciudad Universitaria D.F.,México 04510 Mexico; 3 Center of Biotechnology Research. Autonomous University of Morelos. Mexico

## Abstract

O-Cholesteryl-O-phenyl-N-phenylphosphoramidate (**1**) and four organotin (lV) derivatives of the ambidentate *O*-cholesteryl-*O* -phenyl phosphorothioate ligand formulated as Me_3_ SnOSPR’R”(**2**), Ph_3_SnOSPR’R”(**3**), O(CH_2_CH_2_S)_2_Sn(*n*-Bu)OSPR’R”(**4**), S(CH_2_CH_2_S)_2_Sn(*n*-Bu)OSPR’R”(**5**), (R’ = *O*-phenyl; R”= *O*-cholesteryl) were subjected to cytotoxicity screening against KB (nasopharingel carcinoma), OVCAR-5 (ovarium carcinoma) and SQC-1 UlSO (squamous cell cervix carcinoma) cell cultures. The results of the bioassay showed that these compounds possess potent antitumor activities against the studied human carcinoma cell lines.


### CYTOTOXIC ACTIVITIES OF O-CHOLESTERYL-O-PHENYL-N-PHENYL-PHOSPHORAMIDATE AND ITS ORGANOMETALLIC TIN(IV) DERIVATIVES

Marcela López-Cardoso,[1] Patricia García y García,[1] Raymundo Cea-Olivares*,[2] and María- Luisa Villareal.[3]

[1] Center of Chemical Research. Autonomous University of Morelos
Av. Universidad 1001, Cuernavaca, 62210, Morelos, México.
[2] Institute of Chemistry, National Autonomous University of México, Circuito Exterior, Ciudad Universitaria, México, 04510, D.F., México. E-mail: cea@servidor.unam.mx
[3] Center of Biotechnology Research. Autonomous University of Morelos.

**ABSTRACT**

O-Cholesteryl-O-phenyl-N-phenylphosphoramidate **(1)** and four organotin (IV) derivatives of the ambidentate *O*-cholesteryl-*O*-phenyl phosphorothioate ligand formulated as $Me_3SnOSPR'R''$**(2)**, $Ph_3SnOSPR'R''$**(3)**, $O(CH_2CH_2S)_2Sn(n\text{-}Bu)OSPR'R''$**(4)**, $S(CH_2CH_2S)_2Sn(n\text{-}Bu)OSPR'R''$**(5)**, (R'= *O*-phenyl; R"= *O*-cholesteryl) were subjected to cytotoxicity screening against KB (nasopharingel carcinoma), OVCAR-5 (ovarium carcinoma) and SQC-1 UISO (squamous cell cervix carcinoma) cell cultures. The results of the bioassay showed that these compounds possess potent antitumor activities against the studied human carcinoma cell lines.

### INTRODUCTION

Phosphoramidates have considerable biological interest as anticancer agents [1], and have been widely used, according to the scheme 1 [2], in the synthesis of organophosphates, phosphorothioates and phosphoroselenoates. We have prepared and structural characterized the two epimeric of O-Cholesteryl-O-phenyl-N-phenylphosphoramidates **(1)**, that were used for the preparation of a new ambidentate phosphorothioate ligand containg a O-cholesteryl residue [3].

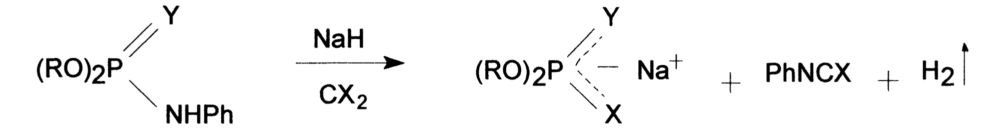

X = O, S, Se ; Y = O, S, Se

Scheme 1: Synthesis of phosphates, phosphorothioates and phosphoroselenoates.

The organotin derivatives with natural products have received considerable impulse by inorganic and organometallic chemist for diverse reasons, including the potential bioactivity. The organotin(IV) derivatives with steroids exhibit biological activities (especially antitumour) [4-6] and have been studied with respect to the reactivity and interesting structural features. The structural chemistry of phosphorus-based organo tin(IV) derivatives is influenced by the number and nature of organic groups bonded to tin and phosphorus atoms, as well as the type of donor atom (O, S, Se) [7]. Recently, we have reported the preparation of a new ambidentate phosphorothioate ligand, containing a bioactive group, Na[OSPR'R"], [R'= *O*-Phenyl, R"= *O*-cholesteryl] [8], that was used for the synthesis of four organometallic tin (IV) **(2-5)** [9]. In this paper, we report the results of the bioassay of compounds **(1-5)**, showing that they posses potent antitumor activities against KB (nasopharyngel carcinoma), OVCAR-5 (ovarium carcinoma) and SQC-1 UISO (squamous cell cervix carcinoma).

### MATERIALS AND METHODS

Preparation and spectroscopic features of the pure compounds tested in this work **(1-5)** were reported previously [3, 9], they were characterized by elemental analyses, IR, MS and multinuclear NMR ($^1H$, $^{13}C$, $^{31}P$, $^{119}Sn$) spectroscopy. The structures of these compounds are shown in scheme 2.

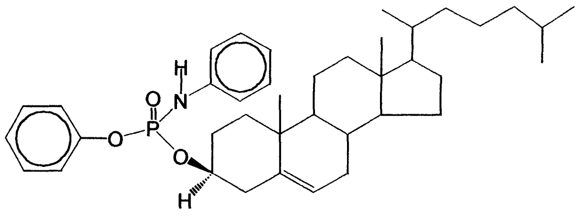

**(1)**

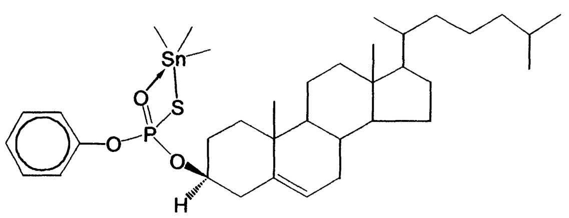

**(2)**

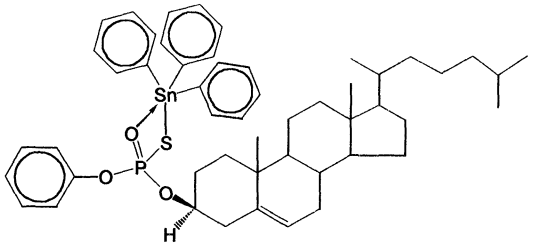

**(3)**

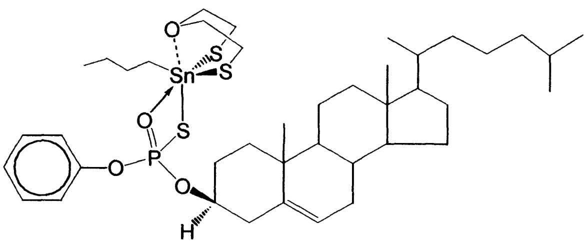

**(4)**

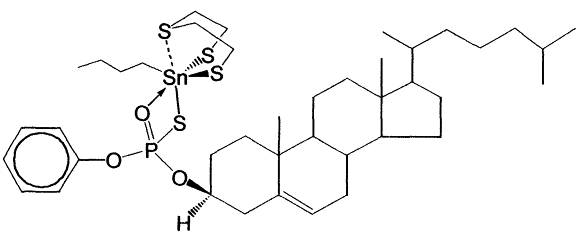

**(5)**

*Scheme 2:* O-cholesteryl-O-phenyl-N-phenylphosphoramidate **(1)** and the four Organotin (IV) derivatives of the ambidentate *O*-cholesteryl-*O*-phenyl phosphorothioate ligand **(2-5)**.

*Evaluation of Biological Activity. Cytotoxic Activity.*

The KB (nasopharyngel carcinoma), Ovcar-5 (ovarium carcinoma), and SQC-1 UISO (squamous cell cervix carcinoma) cell lines were maintained in BME (Basal Medium Eagle) with 10% fetal bovine serum (FBS). All cell lines were cultured at 37 °C in an atmosphere of 5% $CO_2$ in air (100% humidity).

The cells at a log phase of their growth cycle were treated in triplicate at various concentrations of the pure compounds to evaluate (0.5-100 μg/ml), and incubated for 72 h at 37 °C in a humidified atmosphere of 5 % $CO_2$. The cell concentration was determined by protein analysis. Results were expressed as the dose that inhibits 50% control growth after the incubation period (**$ED_{50}$**). The values were estimated from a semilog plot of the drug concentration (μg/ml) against the percent of viable cells. Pure compounds with $ED_{50} \leq 4$ μg/ml were considered active according to National Cancer Institute (NCI) guidelines described in the literature [10] (Geran et al., 1972).

### RESULTS AND DISCUSSION.

*Cytotoxic Activities*

Table 1 shows the cytotoxic data of the tested pure compounds (**1-5**). This compounds containing O-cholesteryl, show significant cytotoxic activity towards SQC-1 UISO cell line ($ED_{50}$ values 1.7, <1, <1, 1.5, <1 μg/mL, respectively). They also showed a moderate toxicity against KB cell line ($ED_{50}$ values 3.3, >4, 2.7, 3.1, 2.8 μg/mL, respectively) and significant and moderate cytotoxic activity against the Ovcar-5 cell line ($ED_{50}$ values <1, <1, 2.9, 2.8, >4 μg/mL, respectively).

According to these results, the five compounds exhibited important cytotoxic activities against the tested cell lines in culture. From the three studied cell lines, the cervix carcinoma SQC-1 UISO was the most sensitive to the action of the compounds under investigation. Compound **2** displayed an important toxic activity against SQC-1 UISO and OVCAR-5, and was inactive toward the nasopharyngel carcinoma KB. In conclusion the results showed that compounds **1-5** presented cytotoxic action towards the studied cell lines.

**Table 1**. Cytotoxicity of pure compounds ($ED_{50}$ (μg/ml)): PhNH(O)PR'R" (**1**), $Me_3SnOSPR'R"$ (**2**), $Ph_3SnOSPR'R"$ (**3**), $O(CH_2CH_2S)_2Sn(n\text{-}Bu)OSPR'R"$ (**4**), $S(CH_2CH_2S)_2Sn(n\text{-}Bu)OSPR'R"$ (**5**). [R'= *O*-Ph, R"= *O*-cholesteryl].

| | **$ED_{50}$ values (μg/ml)** | | |
|---|---|---|---|
| | **Cell line** | | |
| ***Compounds*** | **KB** | **SQC-1 UISO** | **OVCAR-5** |
| **(1)** PhNH(O)PR'R" | 3.3 | 1.7 | < 1 |
| **(2)** $Me_3SnOSPR'R"$ | > 4 | < 1 | < 1 |
| **(3)** $Ph_3SnOSPR'R"$ | 2.7 | < 1 | 2.9 |
| **(4)** $O(CH_2CH_2S)_2Sn(nBu)OSPR'R"$ | 3.1 | 1.5 | 2.8 |
| **(5)**$S(CH_2CH_2S)_2Sn(nBu)OSPR'R"$ | 2.8 | < 1 | > 4 |
| ***Antitumor agents (controls):*** | | | |
| Colchicine | < 1 | 1.58 | < 1 |
| Ellipticine | 1.58 | 1.77 | 0.6 |

### REFERENCES

[1] K. Pankiewicz, R. Kinas, WJ Stec, AB Foster, M. Jarman, JMS Van Maanen *J. Am. Chem. Soc.* **101** (1979) 7712.

[2] WS Wadsworth, WD Emmonss *J. Org. Chem.* **29** (1964) 2816

[3] R. Cea-Olivares, M López-Cardoso, RA Toscano *Monats.. Chemie.* **130** (1999) 1129.

[4] M. Gielen, P. Lelieveld, D. De Vos, H. Pan, R. Willem, M. Biesemans and H. H. Fiebing *Inorg. Chim. Acta.*, **196** (1992) 115

[5] R. Willen, H. Dalil, P. Broekaert, M. Biesemans, L. Ghys, K. Nooter, D. De Vos, F. Ribot and M. Gielen, *Main Group Metal Chem* **535** (1997) 20.

[6] M. Gielen, R. Willen, T. Mancilla, J. Ramharter and E. Joosen, *Silicon, Germanium, Tin and Lead Cmpd.*, **9** (1986) 349.

[7] K. J. Vimal, *Coord. Chem. Rev.* **135/136** (1996) 41

[8] M. López-Cardoso, P. García y García, V. García-Montalvo, R. Cea-Olivares, *Heteroatom Chem.* **11** (2000) 6.

[9] M. López-Cardoso, P. García y García, Alejandro Rogers-Sakuma, R. Cea-Olivares, *Polyhedron* **11** (2000) 1539.

[10] Geran, R. I., N. H., MacDonald M. N., Schumacher A. M., Abbott, B. J. *Cancer Chemother. Rep.* (1972), Part 3(2)

**Received: April 7, 2002 – Accepted: May 8, 2002 –**
**Accepted in publishable format: May 29, 2002**

